# Aluminium Matrix Composite Materials Reinforced by 3D-Printed Ceramic Preforms

**DOI:** 10.3390/ma16155473

**Published:** 2023-08-04

**Authors:** Marek Kremzer, Błażej Tomiczek, Grzegorz Matula, Michał Gocki, Łukasz Krzemiński

**Affiliations:** Scientific and Didactic Laboratory of Nanotechnology and Material Technologies, Faculty of Mechanical Engineering, Silesian University of Technology, Konarskiego 18a St., 44-100 Gliwice, Poland; grzegorz.matula@polsl.pl (G.M.); michal.gocki@polsl.pl (M.G.); lukasz.krzeminski@polsl.pl (Ł.K.)

**Keywords:** metal matrix composite, Fused Deposition Modeling (FDM), gas-pressure infiltration

## Abstract

This article employed the fused deposition modelling (FDM) method and gas-pressure infiltration to manufacture alumina/AlSi12 composites. Porous ceramic skeletons were prepared by FDM 3D printing of two different alumina powder-filed filaments. The organic component was removed using a combination of solvent and heat debinding, and the materials were then sintered at 1500 °C to complete the process. Thermogravimetric tests and DTA analysis were performed to develop an appropriate degradation and sintering program. Manufactured skeletons were subjected to microstructure analysis, porosity analysis, and bending test. The sintering process produced porous alumina ceramic samples with no residual carbon content. Open porosity could occur due to the binder’s degradation. Liquid metal was infiltrated into the ceramic, efficiently filling any open pores and forming a three-dimensional network of the aluminium phase. The microstructure and characteristics of the fabricated materials were investigated using high-resolution scanning electron microscopy, computer tomography, hardness testing, and bending strength testing. The developed composite materials are characterized by the required structure—low porosity and homogenous distribution of the reinforcing phase, better mechanical properties than their matrix and more than twice as high hardness. Hence, the developed innovative technology of their manufacturing can be used in practice.

## 1. Introduction

New lightweight structures, mostly made of light metal alloys, have become increasingly in demand in recent years. Light metal alloys are being applied more and more frequently, particularly in the construction, transportation, and aerospace sectors. These materials must, in particular, possess high strength, sufficient corrosion resistance, and excellent safety coefficients. One of the most important problems in material engineering is the development of desirable materials [1,2]. An effort was made to explain the phenomena that result in materials with suitable characteristics. Consequently, it is important to investigate the impact of the infiltration parameters of powder metallurgy-produced ceramic preforms on the structure and morphology of the precipitates created in the ceramic–metal composites. These criteria significantly influence the characteristics of the final product and all products made from it. Many published works address composites infiltrated with liquid aluminium or aluminium alloy reinforced with various particles, e.g., SiC [3,4], TiC [5], Al_3_Ti [6], and Al_2_O_3_ [7].

The resistance to cracking and fracture toughness of ceramic–metal composite materials are essential characteristics [8]. The mechanical properties of these materials have been improved through various technical approaches. Heat treatment of manufactured composites has been used to increase the metallic matrix’s strength while preserving the ceramic reinforcement’s anti-wear qualities. The technology of liquid metal infiltration into ceramic preforms to enhance composites’ characteristics has recently gained popularity [9,10,11]. The impact of manufacturing factors on the sliding characteristics and abrasion resistance of the examined composites has received attention in the literature [12,13,14]. The best results are obtained utilizing light metal alloys, according to computer modelling studies of the structures and behaviours at the ceramic–metal alloy border [10,11,15,16]. Aluminium alloy-filled components deliver the best performance, and alumina is one of the most widely used ceramic phases in these composites [17,18].

Applying fused deposition modelling (FDM) as a 3D printing method, modern porous materials suitable for pressure infiltration can be produced in this way. Narrow dimensional tolerances can be used to manufacture complex dimensional parts using FDM. 3D printing can be performed in a direct way, where the printout does not require further processing [19,20,21], and indirectly, where it is necessary to use chemical and thermal treatment of element [22,23,24,25]. Additive technologies for the production of ceramic materials, especially SLS, unfortunately, are not developing as rapidly as in the case of metals. This is mainly due to low thermal conductivity and cracking during printing [8,26]. For this reason, a better solution is to print from a slurry containing a large proportion of ceramic particles and then remove the binder and sinter them [22,27,28]. Filaments for FDM printing, highly filled with ceramic particles, have recently appeared on the market. Printed components should be subjected to binder degradation and sintering to achieve a full ceramic or metallic material using an FDM method. For this purpose, chemical and thermal degradation of the binder must be carried out. Due to the risk of cracking, blistering or other material defects, this step is significant and must be carefully optimised [29]. Manufacturers of commercial filaments focus on such a selection of the type and shape of binder and size of ceramic particles that show optimal rheological properties during printing (good wettability of ceramics). In addition, the ratio of the polymer and ceramic phases should ensure the lowest possible porosity after sintering the printout [30]. No literature reports on filaments’ design for producing porous ceramic materials have been found.

Modern manufacturing technologies of composite materials based on pressure infiltration of 3D-printed ceramic skeletons offers many technological benefits. The undoubted advantage of this solution is the possibility of designing the proportion of pores, their size and shape, not only at the stage of 3D printing but also during the production of the filament by controlling the share of the binder, which also acts as a pore-forming agent. Thanks to the combination of additive manufacturing technology and liquid metal infiltration, it is possible to produce cermets with a precisely designed structure, which can be homogeneous or gradient. The possibility of creating composite elements exposed to intensive abrasive wear characterized by a plastic metal core is also significant. In addition, the manufactured parts characterized by near-net shape do not require further advanced processing, which means that the developed method is close to zero waste technology.

The aim of the study was to develop and optimize the manufacturing technology of modern infiltrated composite materials with a matrix of aluminium alloys reinforced with 3D-printed ceramic skeletons.

## 2. Materials and Methods

This work produced composite materials obtained by pressure infiltration with AlSi12 aluminium alloy of porous ceramic preforms based on sintered alumina powder produced by FDM printing. Al_2_O_3_ IUPAC powder by EasyChem was combined with a multi-component polymer binder using a Zamak Mercator (Skawina, Poland) mixing machine at a speed of 40 rpm and a screw temperature of 170 °C to form a filament loaded with ceramic particles. Using a Zamak Mercator (Skawina, Poland) laboratory extruder, a filament with a diameter of 2.85 mm was produced from the developed mixture at a temperature of 170 °C and a screw rotational speed of 60 rpm. The binder was made from a blend of high-density polyethene (HDPE), acrylonitrile-butadiene-styrene terpolymer (ABS), and ethylene-vinyl acetate copolymer (EVA). The composition of the developed filaments is presented in Table 1.

This polymer combination offers a reasonably low binder viscosity and the possibility of employing acetone as a solvent during one of the stages of polymer binder degradation. Solvent degradation in acetone removes a part of the polymeric material from which the samples are made. Reducing the polymeric part of the material should allow for a more efficient thermal degradation process and the formation of open pores throughout the material. The solvent was selected based on the solubility parameters of ABS and acetone, calculated according to the Van Krevelen theory. The EVA copolymer provides flexibility to the produced filament. At the same time, the high-density polyethene acts as the skeletal polymer, which maintains the shape of the sample in the early stage of thermal degradation. As a result of thermal debinding, oxidation of the macromolecular substances contained in the material occurs. The gaseous products of this process form spherical bubbles located inside the material [30,31].

The selection of components for the polymer blend resulted from detailed experimental studies. Preparation of the optimal composition required analysis of the physicochemical properties of each polymer. Ultimately, two materials with optimal properties were obtained thanks to the conducted research. A commercial filament “Spectrum Alumina” by Spectrum Filaments filled with Al_2_O_3_ powder was also used for comparison purposes.

**Figure 1:**
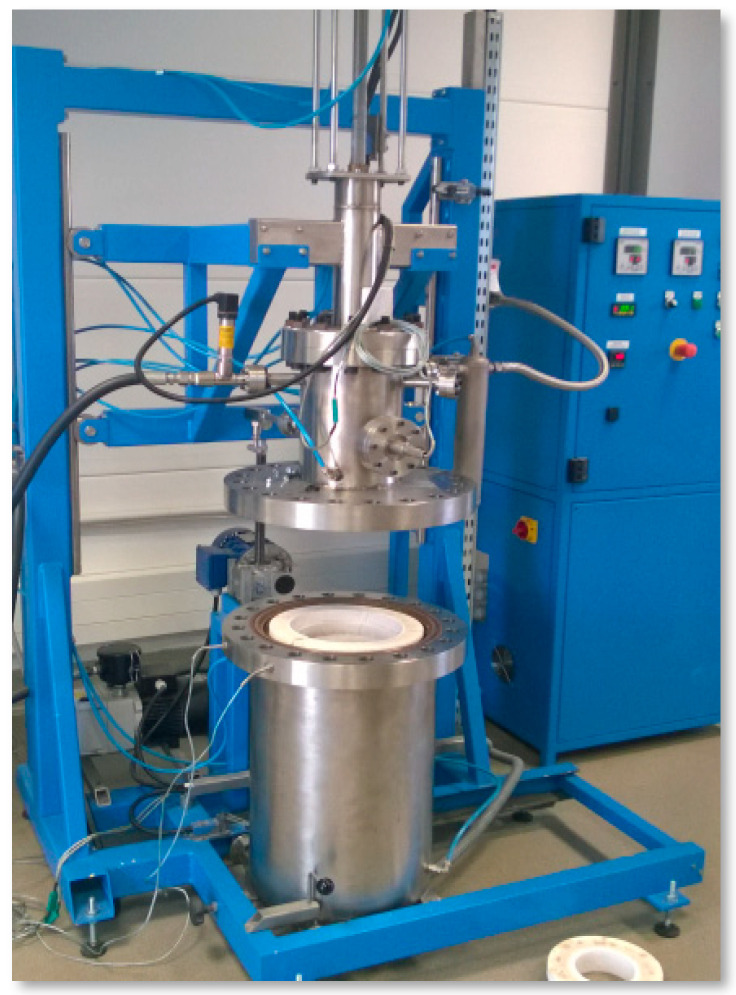
A view of a device intended to infiltrate a porous ceramic material under gas pressure.

Samples with dimensions of 55 mm × 10 mm × 4 mm were printed on a BCN3D Sigma D25 printer. The printing speed was 30 mm/s, the bed temperature was 60 °C, while the nozzle temperature was 205 °C for the commercial Spectrum filament and 240 °C for the one developed in this work. The samples were then put through a debinding procedure involving solvent and thermal treatments. Solvent degradation in acetone was used to partially dissolve the binder and open pores in the material. Thermogravimetric analysis (TGA) was used to experimentally choose the degradation and sintering temperatures for each filament independently. The beginning and final decomposition temperatures of the materials utilized in this investigation had to be determined using the TGA. The heat treatment of samples consists of thermal degradation of the polymer binder and sintering of the Al_2_O_3_ powder. The temperature course of the process consisted of slow heating at the rate of 50 °C/h to the temperature of 600 °C for complete thermal degradation of the organic components, heating at the speed of 300 °C/h to the temperature of 1500 °C and sintering for 1 h. Then, under a nitrogen atmosphere and a pressure of 3 MPa, the created porous ceramic skeletons were infiltrated with an alloy of AlSi12 under gas pressure. Using a PTA-8/PrGC2P device made by the CZYLOK company, the infiltration procedure was carried out at 800 °C for 180 s (Figure 1). The spontaneous saturation proved ineffective due to the ceramic skeleton’s low ability to become wet due to the type of alloy employed and it having the largest percentage of tiny pores. It was decided to raise the infiltration pressure to 3 MPa as a result.

An analysis of the manufactured filaments’ chemical degradation was performed as part of the study. To consider the process of dissolving polymers in liquids, it is necessary to follow this process step by step. After immersing the polymeric material in the solvent, the slow diffusion of liquid molecules into the interior of the polymer begins. As a result of this phenomenon, the material expansion—its dimensions increase. The reason for the increase in the volume of the polymer is the spreading of the macromolecular chains and their solvation. In the next stage, the chains of macromolecules are separated, and the good solubility of the polymer begins. The dissolution process occurs spontaneously when it satisfies the thermodynamic relationship. The thermodynamic potential change detected 32, 33, 34. Δ*G* must be negative. The change in this potential is closely related to enthalpy (Δ*H*) and entropy (Δ*S*). The relationship of these three values is shown in Equation (1).

Δ*G =* Δ*H* − *T*∙Δ*S* (1)

The most complete systematics regarding the relationship between the molecular structure of substances and their solubility was formulated in 1976 by an outstanding Dutch chemist—Dirk Willem van Krevelen. Van Krevelen distinguished three components of the solubility parameter for each functional group and other atoms that make up a given chemical compound. The first component is associated with dispersion forces (*δ_d_*^2^), the second with polar forces (*δ_p_*^2^), and the third component is related to the formation of hydrogen bridges (*δ_h_*^2^). The following formula can be used to determine the solubility parameter of both the polymer and its solvent [31,32,33,34,35]:

(2) $$\delta^{2}=\delta_{d}^{2}+\delta_{p}^{2}+\delta_{h}^{2}$$

The values of individual components are calculated from the formulas:

(3) $$\delta_{d}=\frac{\sum F_{di}}{V}$$

(4) $$\delta_{p}=\frac{\sqrt{\sum F_{pi}^{2}}}{V}$$

(5) $$\delta_{h}=\sqrt{\frac{\sum E_{hi}}{V}}$$

where: *F_d_*—component of dispersion forces, *F_p_*—component of polar forces, *E_h_*—component of hydrogen bonding forces, *V*—molar volume.

**Figure 2:**
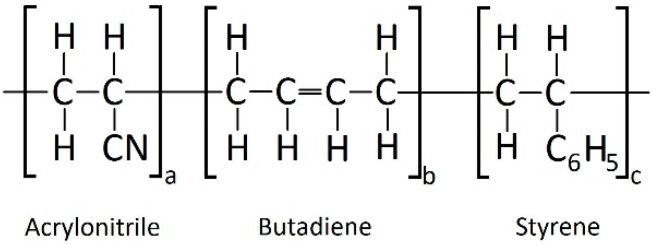
Structural formula of terpolymer (ABS).

In this study, chemical degradation of the polymeric material was carried out based on the solubility of polymers in organic solvents. One of the polymer components in the prepared powder-polymer slip was acrylonitrile-butadiene-styrene terpolymer (ABS). The structural formula of terpolymer (ABS) is presented in Figure 2.

According to the physicochemical calculations, the solubility coefficient for this polymer material is 18.88 J^1/2^ cm^3/2^. In light of the analysis of individual solubility coefficients of organic solvents, acetone was selected as the appropriate liquid for the process of chemical degradation of ABS material. Acetone has a solubility coefficient of 20.3 J^1/2^ cm^3/2^. The difference between the coefficients in the tested system was 1.42 J^1/2^ cm^3/2^, which means that, according to van Krevelen’s theory, the material will dissolve in the tested solvent.

To perform empirical tests, samples of filament filled with 30% and 25% Al_2_O_3_ powder were produced on a 3D printer. The samples were cube-shaped, and their exact dimensions are presented in Table 2.

Due to the fact that the only component of the polymer-powder slurry that dissolved in acetone was ABS terpolymer, the weight loss of each sample was converted into a percentage weight loss of the tested polymer. Dynamic thermal analysis (TG, DTG, DTA) of both the Spectrum commercial filament and the filament developed in this work was performed. The tests were conducted in the Netzsch STA 409 PC/PD device with an air environment and a 10 °C/min heating rate up to 600 °C. The weight of the analyzed samples was 100 mg. The temperature measurement was calibrated based on a set of calibration standards produced by NETZSCH no. 6.223.5-91.3. The correctness of mass changes indication was checked with the use of the Leco standard No. 502-09. A scanning electron microscope (SEM), a Zeiss Supra 35, was used to observe the microstructures of both ceramic preforms and composite materials.

The composite material was tested with Nikon X TH 225 ST 2× computed tomography CT. The analysis of porosity and pore distribution was performed in the VGStudio-MAX software. The analysis method was based on the VGEasyPore algorithm of the VGStudioMax software. The algorithm is used to identify voxels based on the current local grey value of the material image in relation to a specific local contrast threshold in the pore boundary area. To determine the porosity, the parameters of the algorithm were established:

− Local contrast threshold in the grey tonal range of the entire projection;
− Range of grey change gradient identification to determine the threshold of contrast changes;
− Pore probability threshold based on the grey gradient of the projection;
− Maximum and minimum pore sizes are included in the statistical analysis.

To determine the mechanical characteristics of ceramic preforms, obtained composite materials, and AlSi12 aluminium alloy (as reference material), three-point bending tests were performed on the SHIMADZU mechanical testing apparatus. The deformation rate was 1 mm/min and the initial force was set to 0.1 N.

The hardness tests of the developed composite materials and the AlSi12 alloy constituting their matrix were carried out using the Rockwell method in the A scale on the Zwick ZHR hardness tester.

## 3. Results

### 3.1. Thermal Analysis of Filaments

**Figure 3:**
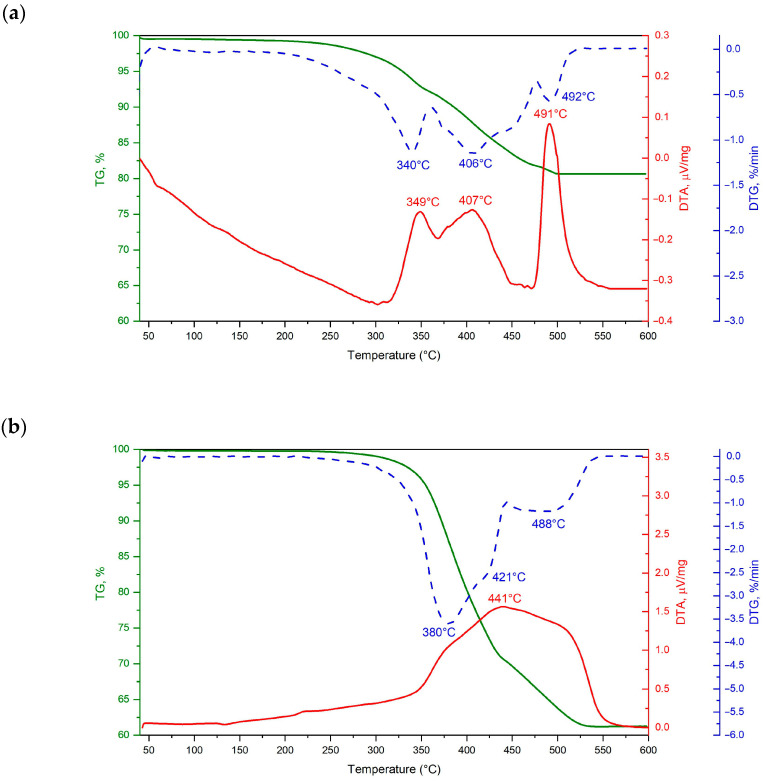
The results of the TGA/DTA analysis of (**a**) Spectrum and (**b**) developed filament.

Due to the increased temperature used at the various stages of manufacturing the designed composite material, the DTA analysis was performed for both filaments (Figure 3). The thermal behaviour of the binder polymer in the filament is essential not only at the printing stage but also during subsequent sintering. The thermoplastic binder must be removed from the components after 3D printing to get a porous ceramic sample. Spectrum and developed filament showed similar thermal degradation behaviour up to 200 °C. After this point, according to the DTG curve for Spectrum filament, three different stages of weight loss were observed. The first stage began above 200 °C and continued to 360 °C. At this stage, a slight weight loss of approximately 8% was observed. Up to 476 °C, the second step of weight loss in the Spectrum filament proceeded. At this point, an additional 10.2% weight loss was seen. The last stage of weight loss, which ends at a temperature of 505 °C for Spectrum filament, causes a loss of 1.2%. Although developed filament showed a relatively steeper weight loss compared to the commercial one. In the case of the DTG curve of the developed filament, the first two stages of weight loss are combined, and their separation is not so apparent. In the range from 200 to 380 °C, the annealed sample loses 12.5% of its mass. The most intense weight loss is observed in the range of 380 to 42 °C, where the developed filament loses almost 12.7%. The overall mass loss calculated up to 488 °C was about 35%. The last weight loss of 4.2% was set for the stage ending at 530 °C. Again, the developed sample showed a relatively steeper weight loss compared to the Spectrum filament. In total, the mass loss of the developed filament is much higher and reaches as much as 39.7%. This is, of course, due to twice the content of polymeric binder compared to the Spectrum filament, where the decrease is only 19.4%. The DTA diagram of Spectrum filament shows three characteristic peaks: 340, 406 and 492 °C that correspond to the individual components of the binder used in the filament. In the case of the developed filament, these characteristic peaks are 380, 420 and 488 °C. In the beginning, around 120 °C and 220 °C, a slight distortion of the curve is also visible.

### 3.2. Chemical Degradation Analysis

The results of the chemical degradation analysis of the developed filaments are presented in Table 3. Because the only component of the polymer-powder slurry that dissolved in acetone was ABS terpolymer, the weight loss of each sample was converted into a percentage weight loss of the tested polymer. As a result of the analysis of Dirk W. van Krevelen’s theory and empirical research, it should be concluded that the chemical degradation process of the tested material was correct. ABS polymer degradation occurred in the material filled with 30% and 25% Al_2_O_3_ powder. The average weight loss of the polymer in the sample filled with 30% ceramics was about 22% by mass, while in the sample filled with 25%, only 12% of the mass of the polymer was dissolved. This relationship may be due to the greater amount of polymers insoluble in a given solvent in a sample containing 25% alumina. A larger amount of the EVA polymer was homogenized with the ABS polymer and prevented the solvent from accessing the chains of the terpolymer in question.

### 3.3. Microstructure Observations

**Figure 4:**
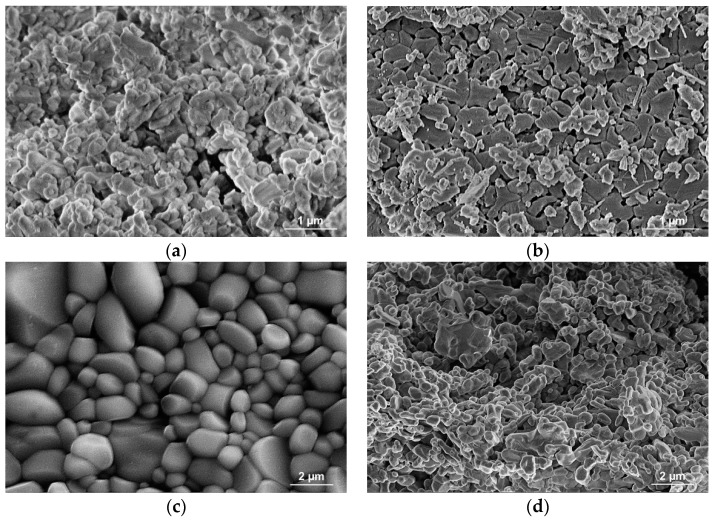
SEM images of (**a**) Spectrum Al_2_O_3_ powders, (**b**) EasyChem Al_2_O_3_ powder, (**c**) fracture of porous ceramic skeleton obtained on the base of Spectrum filament, and (**d**) fracture of porous ceramic skeleton obtained on the base of developed filament.

Figure 4 shows the results of observations of Al_2_O_3_ powders made in a scanning electron microscope (SEM). The ceramic powder used to manufacture the Spectrum filament was obtained by thermally degrading the filament binder (Figure 4a). Figure 4b shows the Al_2_O_3_ powder used to make our own filament. The particles of both powders are submicron in size with irregular shapes. The porous sintered ceramics’ fracture surface analysis didn’t reveal any obvious structural defects like fractures, gas bubbles, or pore clusters that might appear after the breakdown of the polymeric binder. Comparing the morphology of sintered ceramics (Figure 4c,d) can be observed that in the case of Spectrum filament, sintering temperature causes reduce the particles’ surface through their sferoidization, smoothing the surface and bonding the particles. The morphology of the ceramic preform’s fractures was examined, and the results showed that sintering causes the powder particles to grow and densify (Figure 4c). In the case of skeletons based on the developed filament, there was no growth and densification of ceramic particles because this filament was characterized by twice as much organic content as Spectrum (approx. 40% by mass compared to 20% in the case of Spectrum), which prevented the particle from approaching the distance that guaranteed mass flow (Figure 4c).

**Figure 5:**
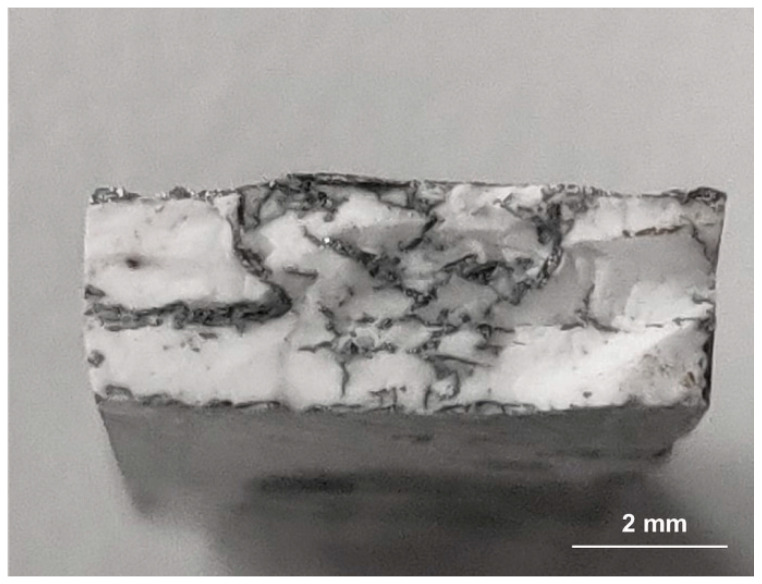
Fracture of a preform based on Spectrum filament infiltrated with aluminium alloy.

Based on geometrical measurements and the weight of the developed frameworks, with the known density of Al_2_O_3_, the porosity of the produced ceramic frameworks was estimated. It is 29% for semi-finished products based on the Spectrum filament and 71% and 68%, respectively, for preforms based on the manufactured filament with 25% and 30% of aluminium oxide powder. The low porosity of the skeletons based on the commercial filament is due to the low proportion of organic components, reaching 20% by weight. This results in the fact that these materials were infiltrated with aluminium alloy only in the areas of structure defects (Figure 5). The poor infiltration of the material excluded it from further research.

**Figure 6:**
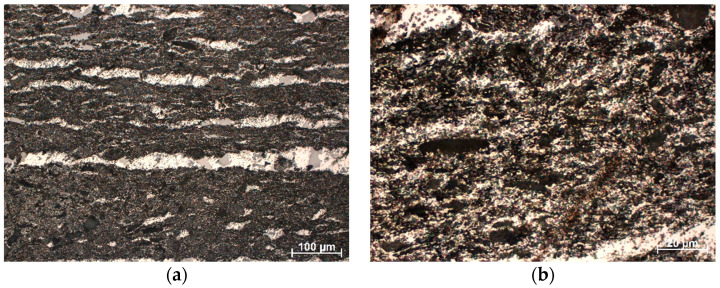
Microstructure of infiltrated composites reinforced with porous ceramic manufactured on the base of filament with 30% Al_2_O_3_: (**a**) magnification 200× and (**b**) magnification 1000×.

As a result of light microscope examinations at a magnification of 200×, parallel channels filled with aluminium alloy were observed (Figure 6a). These channels were formed during the additive manufacturing of the ceramic frameworks and constituted the spaces between the layers of the print. The fine-grained structure of the composite material and even distribution of the reinforcing phase in the alloy matrix were observed in micro-areas between channels at 1000× magnification (Figure 6b). In the image, the light areas are the alloy, and the black sites are ceramic.

**Figure 7:**
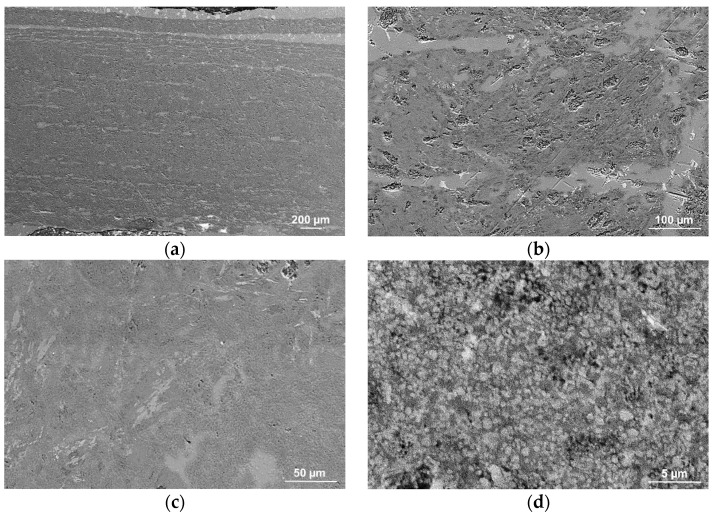
SEM images of infiltrated composites reinforced with porous ceramics sinters: (**a**) magnification 75×, (**b**) magnification 500×, (**c**) magnification 1000×, and (**d**) magnification 10,000×.

The resulting composites’ lack of considerable porosity and effective filling of the ceramic preform by aluminium alloy were revealed by SEM image evaluation. In addition, channels created between the printing layers were tightly filled with liquid metal (Figure 7a,b). Microfiltration into the ceramic matrix was discovered by a more thorough examination (Figure 7c,d), which enables a strong bond between the ceramic and metallic phases and permits good mechanical qualities. Investigations verified the normal distribution of sintered alumina particles, the microstructure’s percolation type, and the absence of reinforcement agglomeration and unfilled pores. Ceramic grains kept their form, suggesting the infiltration pressure was appropriate. The metallic matrix and grains are also in close contact, which helps to achieve good mechanical characteristics and demonstrates that the infiltration process was carried out at an optimum temperature.

**Figure 8:**
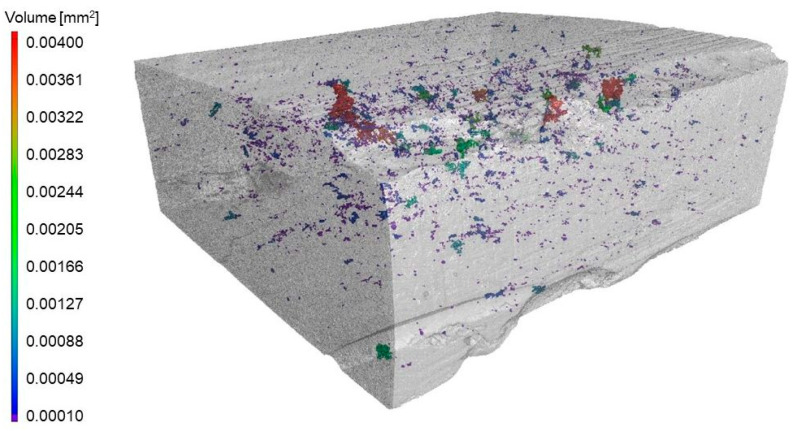
Pores distribution in composite material reinforced with preform manufactured on the base of filament with 30% portion of the ceramic phase.

As a result of the analysis of the porosity of the composite material reinforced with a skeleton based on the developed filament, it was found that it is only 0.07%. The largest identified pore formed due to joining small voids was 0.007 mm^3^. Only nine pores have a volume exceeding 0.002 mm^3^, while most pores (over 90%) do not exceed 0.0005 mm^3^. The distribution of pores in the tested material is shown in Figure 8.

### 3.4. Mechanical Properties

**Figure 9:**
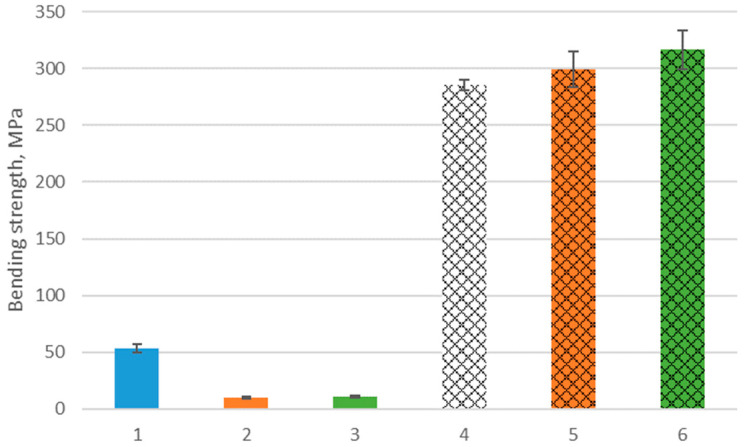
The bending strength of 1. Preform on the base of Spectrum filament, 2. Preform on the base of obtained filament with 25% of Al_2_O_3_, 3. Preform on the base of obtained filament with 30% of Al_2_O_3_, 4. AlSi12 alloy, 5. Composite on the base of preform “2”, and 6. Composite on the base of preform “3”.

A static bending test was conducted to determine the strength of the provided matrix’s AlSi12 alloy, composite components, and ceramic preforms. AlSi12 alloy has a 285.2 MPa average bending strength under delivery conditions. A bending strength of 53.4 MPa characterizes the ceramic preforms obtained on the base of Spectrum filaments, while semi-finished products made based on the developed filament (with 30% volume fraction Al_2_O_3_) show bending strength at 11.2 MPa. The composite material obtained based on their pressure infiltration is characterized by the highest bending strength of 316.7 MPa, which is an 11% increase in relation to its matrix. The material reinforced with a preform made of a filament with a 25% volume portion of the ceramic phase was characterized by a bending strength of 299.7 MPa. The bending strength results are shown in Figure 9.

**Figure 10:**
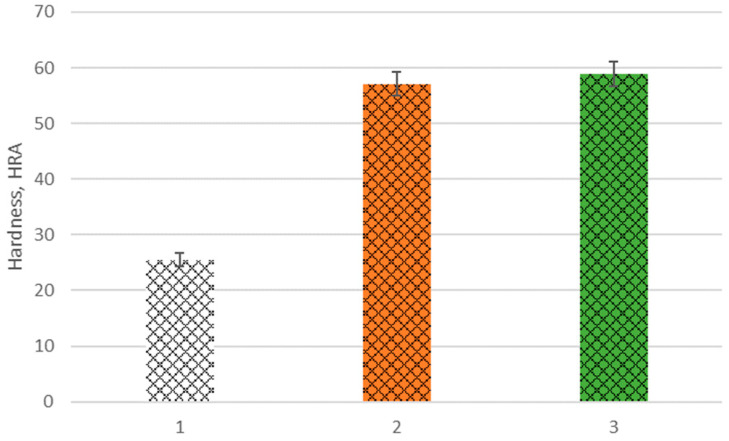
The hardness of: 1. AlSi12 alloy, 2. Composite material reinforced by preform on the base of filament with 25% of Al_2_O_3_, and 3. Composite material reinforced by preform on the base of filament with 30% of Al_2_O_3_.

The results of hardness measurements of the developed composites and their matrix are shown in Figure 10. The highest hardness among the tested materials of 58.9 HRA is characterized by a composite reinforced with the preform made of the developed filament with a 30% share of Al_2_O_3_ powder. The hardness of this material is more than two times higher than its matrix—AlSi12 alloy, whose is 25.2 HRA. The composite reinforced with a skeleton printed with a filament with 25% of ceramic powder has a hardness of 57.1 HRA.

## 4. Discussion

A well-manufactured ceramic preform should have a high permeability value and a structure of open and connected pores and channels. Sufficiently high permeability is not ensured by submicron interconnected pores because even when their share is significant, they cause high flow resistance. According to SEM observations of the fracture morphology, the desired structure has only been obtained in the case of skeletons built using the filament created as part of the project. This is seen by the pores that develop due to the polymeric binder’s decomposition, which does not disappear during sintering. The distribution and shape of the pores do not show a preferential arrangement concerning the printing direction, which is typical, for example, for uniaxial pressing [36]. Preforms printed with Spectrum filament are characterized by high density resulting from the low portion of organic material in the filament (approximately 20%). Such a situation is conducive to the approach of the ceramic particles at a distance that allows mass transport and, as a result, rounding and growth of the ceramic particles. It was tested that the pore content is from about 68% to about 71% in the case of preforms based on developed filaments and 29% in the issue of semi-finished products based on commercial Spectrum filament. The results reported for porosity are consistent with those of earlier studies [37,38].

Printed samples from filament filled with ceramic powder are only semi-finished products. The thermoplastic binder must be removed from the samples after 3D printing to achieve a fully porous ceramic part. The temperature at which individual binder components start to degrade thermally is crucial. Thermogravimetry analysis of filled filaments is necessary because the filler shifts the decomposition temperature to a higher temperature [39]. The decision to base it on the TGA/DTA curve is significant for many reasons. The first is the slurry blending, filament extrusion and FDM printing conditions. In the case of HDPE, partial crosslinking or degradation are potential consequences of treating materials at excessively high temperatures [40]. At the same time, it is important to properly select the conditions for thermal degradation of the binder, i.e., heating rate, time, temperature and atmosphere. When analyzing the DTA curve, individual peaks associated with the exo/endo thermal effect can be seen. Unfortunately, reliable identification is complicated due to the overlapping thermal effects of various polymeric binder components.

Thermal degradation of one of the binder components starts at a temperature of 200 °C and ends at a temperature of 500 °C or higher. Since this binder intends to maintain the shape at a high temperature, the value should be close to the sintering temperature. This is useful in the case of printed parts [41]. Beginning around 120 °C, there is a slight distortion of the curve connected to the ABS glass transition. On the other hand, the second small peak, about 220 °C, can be attributed to an initial oxidation of polyethene. After an initial stage of oxidative induction, the polyetylene component is degraded by an oxidation reaction. A TGA detector can identify weight loss when the primary polymeric chains are broken down, and the weight of the decomposition byproducts is light enough and volatile enough [42]. EVAs are stable up to 300 °C, and rapid weight loss is seen up to the first peak at 380 °C. In the first stage, acetate groups of EVAs are thermally decomposed into volatile acetic acid. The process is called deacetylation [43]. EVA’s first-stage elimination reaction is also called acetic acid shelf [44]. The second region of weight loss can be attributed to polymer chain scission and evaporation. Subsequent rapid weight loss starts after 450 °C due to the splitting of the main hydrocarbon chain [45]. The thermogravimetric test results demonstrate a wide range of binder degradation, with slight variation when employing different filaments. Because various polymers degrade at different temperatures, allowing decomposition during heating, a multi-component binder system is preferred during thermal degradation [46]. The use of the own filament tends to be more attractive due to the higher amount of binder, which effect in higher porosity of sintered components. In the case of alumina powder, using a protective atmosphere is unnecessary. Keeping the shape of the created element at the sintering temperature advances the end of degradation to a lower temperature, which is desirable. The choice of a multi-binder system in both cases allowed to obtain a non-cracked, defect-free, good-quality ceramic material during degradation. A binder made of multiple polymers may make the process of effective decomposition easier and even faster [47,48].

The analysis of the chemical degradation of the developed filament confirmed the correctly selected composition of the organic multi-binder system. Dissolving ABS in acetone led to the formation of pores in the material, which allowed the free escape of gases during the subsequent thermal degradation of the organic binder and prevented the formation of bubbles and cracks in the material. The remaining non-chemically degraded polymers ensured further consistency of the prints. The composition of the multi-binder system provided the required flexibility of the filament and optimal rheological properties during printing [49]. They enabled the sintering of the ceramic phase after its degradation. It can additionally act as a pore-forming agent [50]. A similar two-stage debinding process is presented in many scientific works, where thermal degradation is preceded by chemical dissolution of organic fractions in acetone [12,29], water [23], or catalytically removed [31], which is recommended by BASF for their filled filaments, e.g., Ultrafuse 316LX.

The developed composite material (except the channels formed between the print layers) is characterized by a homogeneous fine-grained structure. Metallographic tests proved that infiltration with liquid aluminium alloy was complete. The fine pores around single ceramic particles were also filled. This condition was also confirmed by tomographic tests with porosity analysis, which was determined at 0.07%. Such results prove that the sintered ceramic skeletons manufactured by the additive technology are characterized by the intended structure of open interconnected pores (high permeability) [5,6].

Composite materials manufactured by pressure infiltration of the printed ceramic preform are characterized by higher bending strength than their matrix, a cast aluminium alloy AlSi12. In addition, an over two-fold increase in the hardness of the developed material in relation to its matrix was observed. Such high mechanical properties prove a good connection between the ceramic and metallic phases of the composite. Commercial filaments filled with ceramic particles are designed to produce solid materials from them, show high compaction during sintering and are not suitable for creating porous structures. In work [29], it was proved that for the Zetamix-Alumina filament (with a similar portion of binder and particle size to Spectrum) at the sintering temperature of 1550 °C, it is possible to obtain a ceramic content exceeding 99%. Because of the above, it was advisable to develop a composition and produce a filament based on which it was possible to achieve the required porosity in the sintered ceramic skeleton.

The developed fabrication technology of composite materials, combining the additive manufacturing of preforms and their pressure infiltration, is characterized by several advantages and technological possibilities, which include, first of all: the production of elements with complex shapes and the possibility of local changes in the portion of reinforcement in the material, moreover, it ensures the required structure and properties of the composite, hence it can undoubtedly be used in practice.

## 5. Conclusions

- Skeletons based on the developed filaments filled with Al_2_O_3_ particles, characterized by the structure of open, connected pores, can be a reinforcement of composites manufactured by pressure infiltration with light metal alloys.
- Due to a broader range of thermal degradation temperatures, using a multi-component binder shifts the end of degradation to a higher temperature, which is desirable in maintaining the shape of the manufactured element up to the sintering temperature.
- A binder system component such as an ABS that dissolves during solvent degradation ensures open porosity. It allows for the free release of decomposition products of organic fractions during subsequent thermal degradation.
- The manufactured composite materials have higher bending strength and hardness than the unreinforced casted aluminium alloy AlSi12.
- Thanks to the combination of additive manufacturing technology and liquid metal infiltration, it is possible to produce composites with a complex geometry and a precisely designed structure, which can be homogeneous, gradient or locally reinforced.

## Figures and Tables

**Table 1:** The composition of the developed filaments.

Filament	Volume Fraction (V%)			
	Al_2_O_3_	ABS	EVA	HDPE
No.1	25	30	30	15
No.2	30	30	25	15

**Table 2:** Dimension of samples used for chemical degradation.

Dimension of Samples [mm]			
Sample	x	y	z
A30%	10.00	10.07	3.79
B30%	9.99	9.95	4.63
C30%	9.92	9.86	5.69
A25%	9.99	9.91	3.88
B25%	10.00	9.92	4.7
C25%	10.19	10.04	5.81

**Table 3:** Weight loss of abs during chemical degradation.

Al_2_O_3_ Portion	30%			25%		
Mass of React Pollymer	m1 (ABS)	m2 (ABS)	Weight Loss %	m1 (ABS)	m2 (ABS)	Weight Loss %
A	0.0958	0.0784	18.1697	0.0845	0.0719	14.9123
B	0.1090	0.0849	22.1163	0.1012	0.0885	12.5468
C	0.1213	0.0887	26.8799	0.1278	0.1159	9.3085

m1—mass befeore degradation, m2—mass after degradation.

## Data Availability

Not applicable.
